# New-Onset Systemic Lupus Erythematosus Complicated by Preeclampsia, HELLP Syndrome, and Lupus Nephritis During a Third-Trimester Pregnancy: A Case Report

**DOI:** 10.7759/cureus.97666

**Published:** 2025-11-24

**Authors:** Ava Josephson, Morgan Alexander, Aditi Radhakrishnan, Lisbeth Tolentino-Rodriguez

**Affiliations:** 1 College of Pharmacy and Health Sciences, Campbell University, Buies Creek, USA; 2 School of Public Health, University of Washington, Seattle, USA; 3 Department of Research, Uplift North Hills Preparatory, Irving, USA; 4 Department of Research, Global Health Leaders, Gilbert, USA

**Keywords:** autoimmune disease, hellp syndrome, high-risk obstetrics, lupus nephritis, preeclampsia, pregnancy, systemic lupus erythematosus

## Abstract

Systemic lupus erythematosus (SLE) is a chronic autoimmune disease that primarily affects women of reproductive age. Most patients are diagnosed prior to conception; however, new-onset SLE during pregnancy is rare and can mimic common obstetric complications, delaying diagnosis and treatment. Such overlap increases the risk of maternal morbidity and adverse pregnancy outcomes (APOs). A primigravida adolescent in her third trimester presented with severe preeclampsia, HELLP syndrome, and nephrotic-range proteinuria. Postpartum, she developed hemorrhage requiring a modified B-Lynch suture in the ICU, along with vulvar edema, discoid skin lesions, joint pain, and photosensitivity. Laboratory evaluation revealed positive antinuclear antibodies, anti-double-stranded DNA antibodies, hypocomplementemia (characterized by low levels of C3 and C4), and proteinuria, confirming the diagnosis of new-onset SLE with lupus nephritis. The clinical course was managed with corticosteroids, hydroxychloroquine, and supportive therapy. This case highlights the diagnostic complexity of autoimmune disease in pregnancy, particularly when clinical manifestations overlap with obstetric emergencies such as HELLP syndrome and preeclampsia. It underscores the need for early consideration of SLE in atypical or multisystem presentations, prompt biomarker testing, and timely initiation of immunosuppressive therapy. Management in a specialized center, with a multidisciplinary team, is essential to optimizing maternal and neonatal outcomes.

## Introduction

Systemic lupus erythematosus (SLE) is a chronic autoimmune syndrome that most commonly affects women in their reproductive age. SLE incidence rates worldwide are about 50-100 per 100,000 adults [1]. SLE is a multifactorial disease that may be influenced by an individual's genetics, epigenetics, or environment [2]. SLE has a wide variety of clinical manifestations and is commonly accompanied by other comorbidities. Due to the inconsistencies in the disease's presentation, a patient is typically diagnosed with SLE after the onset of symptoms. One study found that the average time a patient waited to be diagnosed with SLE after the first time their symptoms arose was two years [3].

The majority of women with SLE have successful pregnancies; however, it should be noted that women with SLE endure a significantly greater risk for adverse pregnancy outcomes (APOs) than women without the disease. The increase in pregnancy complications for women afflicted with SLE may be as much as four times more likely to develop, with 40-50% of SLE women encountering symptoms during gestation. Additionally, the maternal mortality rate is increased by 20 times compared to non-SLE patients [4]. Among the most frequently occurring symptoms of SLE are fever, fatigue, rash, weight loss, joint pain, and muscle pain. The primary organs that are affected by SLE are the kidneys, lungs, heart, brain, and skin [5]. To prevent APOs like miscarriage, stillbirth, hypertension, and flares, women are advised to plan their pregnancies and ensure that they have not experienced any relapses of SLE symptoms in the previous six months leading up to conception [6].

This multispecialty case outlines an exceptional case of the onset of SLE during the third trimester of pregnancy, accompanied by acute preeclampsia, HELLP (hemolysis, elevated liver enzymes, low platelets) syndrome, and manifestations of discoid and lupus nephritis (LN).

## Case presentation

**Figure 1:**
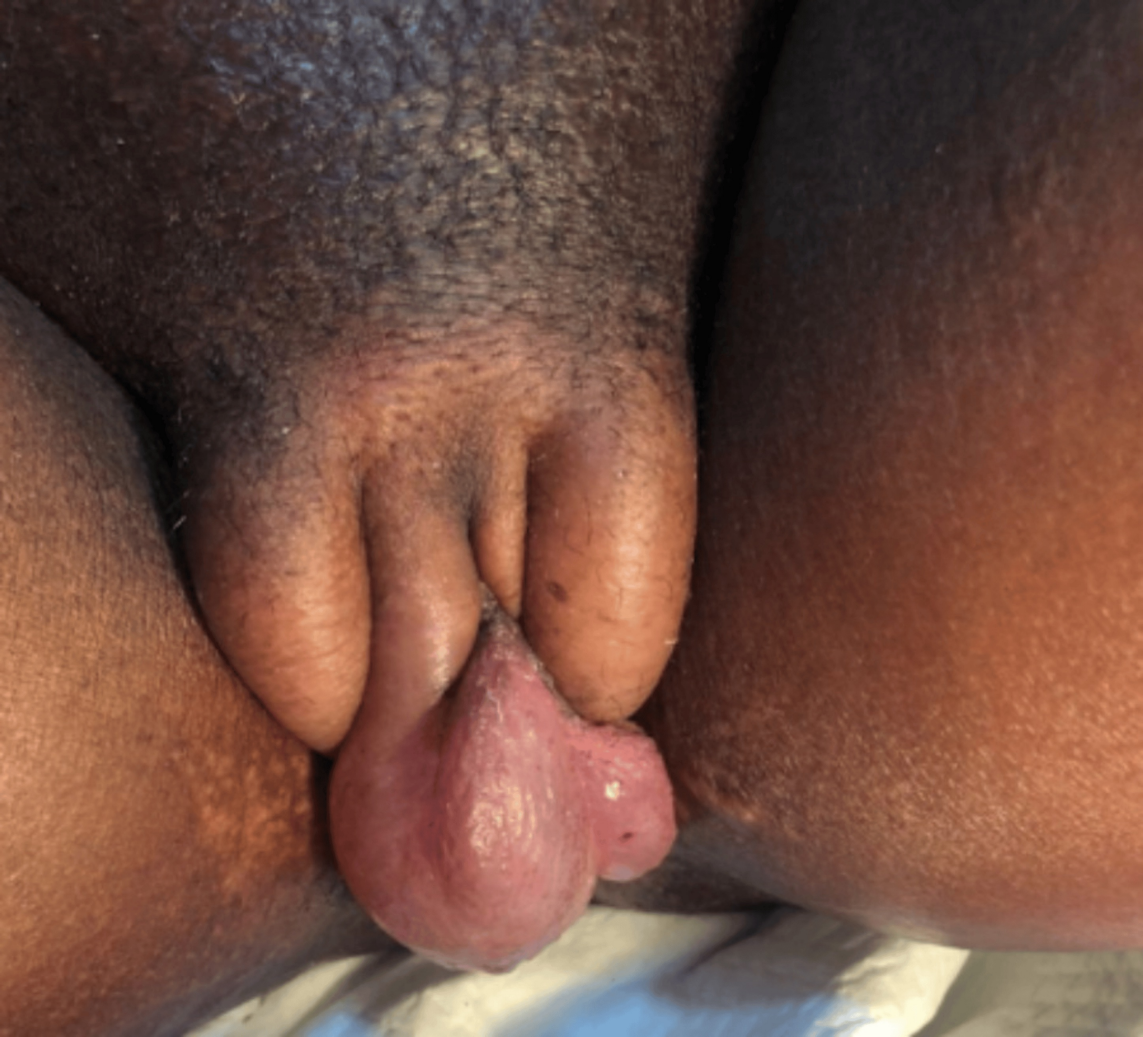
Image of Vulvar Edema Swelling of the patient's vulvar region is shown.

An 18-year-old pregnant woman attending a routine prenatal checkup was admitted to the emergency room due to hypertension and lower limb edema. The patient was at 27.3 weeks of gestation and had attended three previous routine prenatal checkups. She reported this as her first pregnancy and denied toxic habits or relevant family history. On the day of admission, she reported chest pain and oral ulcers and was found to have an oxygen saturation of 74% on room air. After a physical examination, it was revealed that the patient had respiratory distress and decreased breathing sounds bilaterally with crepitus at the left lung base, generalized edema, oral ulcers, and erythematous lesions on the fingertips. Abdominal examination revealed a gravid uterus with fluid waves and tenderness to palpation. A vaginal exam was deferred due to severe vulvar edema (Figure 1).

**Figure 2:**
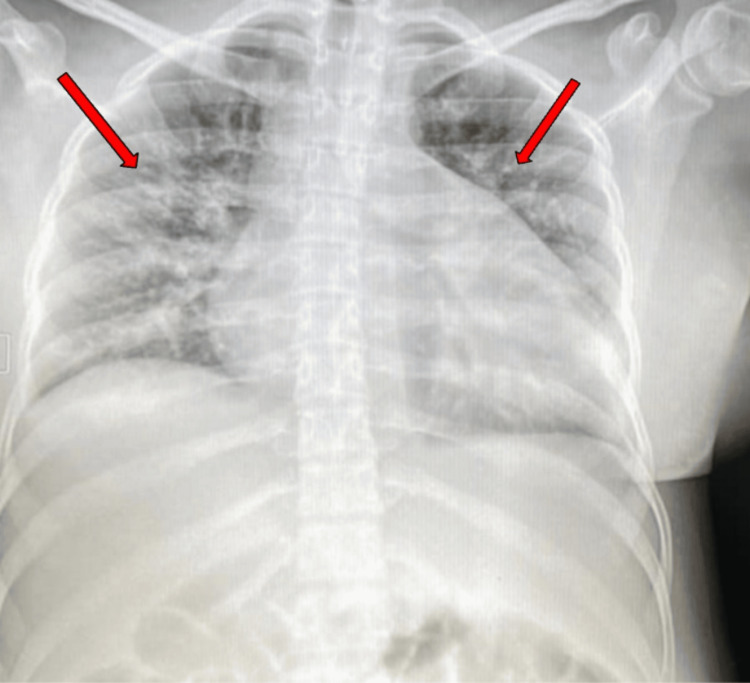
Image of Chest X-ray Fluid accumulation in the patient's chest cavity, regions of interest indicated by arrows.

Vital signs on ICU admission included elevated blood pressure, tachycardia, tachypnea, and hypoxemia. Initial laboratory work revealed anemia, with low hemoglobin and hematocrit levels (Table 1). Imaging studies showed pulmonary edema on chest X-ray (Figure 2) and mild tricuspid regurgitation with posterior pericardial effusion on echocardiogram. Doppler ultrasound ruled out venous thrombosis but noted subcutaneous fluid accumulation. Histopathological evaluation of skin lesions confirmed discoid lupus through evidence of perivascular and interstitial dermatitis with mucin deposition.

**Table 1:** Patient Laboratory Values The patient's laboratory values were collected at three clinical intervals: admission to the ICU, post-surgical, and post-consultation with the nephrology and rheumatology departments. Reference ranges were obtained from Mosby's Diagnostic and Laboratory Test Reference [7]. ALT: aspartate aminotransferase; AST: alanine aminotransferase; LDH: lactate dehydrogenase; g/dL: grams/deciliter; mm^3^: cubic millimeter; U/L: unit/liter.

Test	ICU Admission	Post-Surgical	Post-Consultation	Reference Range
Hemoglobin (g/dL)	8.5	5.4	-	12–16
Hematocrit (%)	26	17	-	37–47
Platelets (/mm^3^)	151,000	126	98,000	150–400
Serum albumin (g/dL)	2.1	2.2	-	3.5–5.5
Total protein (g/dL)	5.3	4.5	-	6.4–8.3
Direct bilirubin (g/dL)	0	0.1	-	0–0.3
Indirect bilirubin (g/dL)	0.3	2	-	0.2–0.8
ALT (U/L)	-	-	95	4–36
AST (U/L)	-	-	82	0–35
LDH (U/L)	-	-	950	100–190

Initial management included magnesium sulfate, antihypertensive therapy, diuretics, corticosteroids for fetal lung maturation, albumin, and antibiotics. A cesarean section was performed at 28 weeks of gestation due to massive vulvar edema, delivering a neonate weighing 1100 grams with an APGAR (appearance, pulse, grimace, activity, respiration) score of 7/8. The complications post-surgery were managed with blood transfusions, a modified B-Lynch suture, and application of a shock suit. The patient also developed steroid-induced hyperglycemia and a second episode of pulmonary edema. Thereafter, the patient attended consultations with the nephrology and rheumatology departments, during which further laboratory testing was performed. The laboratory values following consultation included a reduced platelet count and elevated levels of alanine aminotransferase (AST), aspartate aminotransferase (ALT), and lactate dehydrogenase (LDH). The patient was diagnosed with new-onset chronic SLE complicated with LN, discoid manifestations, severe preeclampsia, HELLP syndrome, and uterine atony with postpartum hemorrhage. The HELLP syndrome diagnosis was supported by thrombocytopenia, elevated liver enzymes, increased indirect bilirubin levels, and a positive direct Coombs test. A summary of the patient's laboratory work used for diagnosis is presented in Table 1.

**Figure 3:**
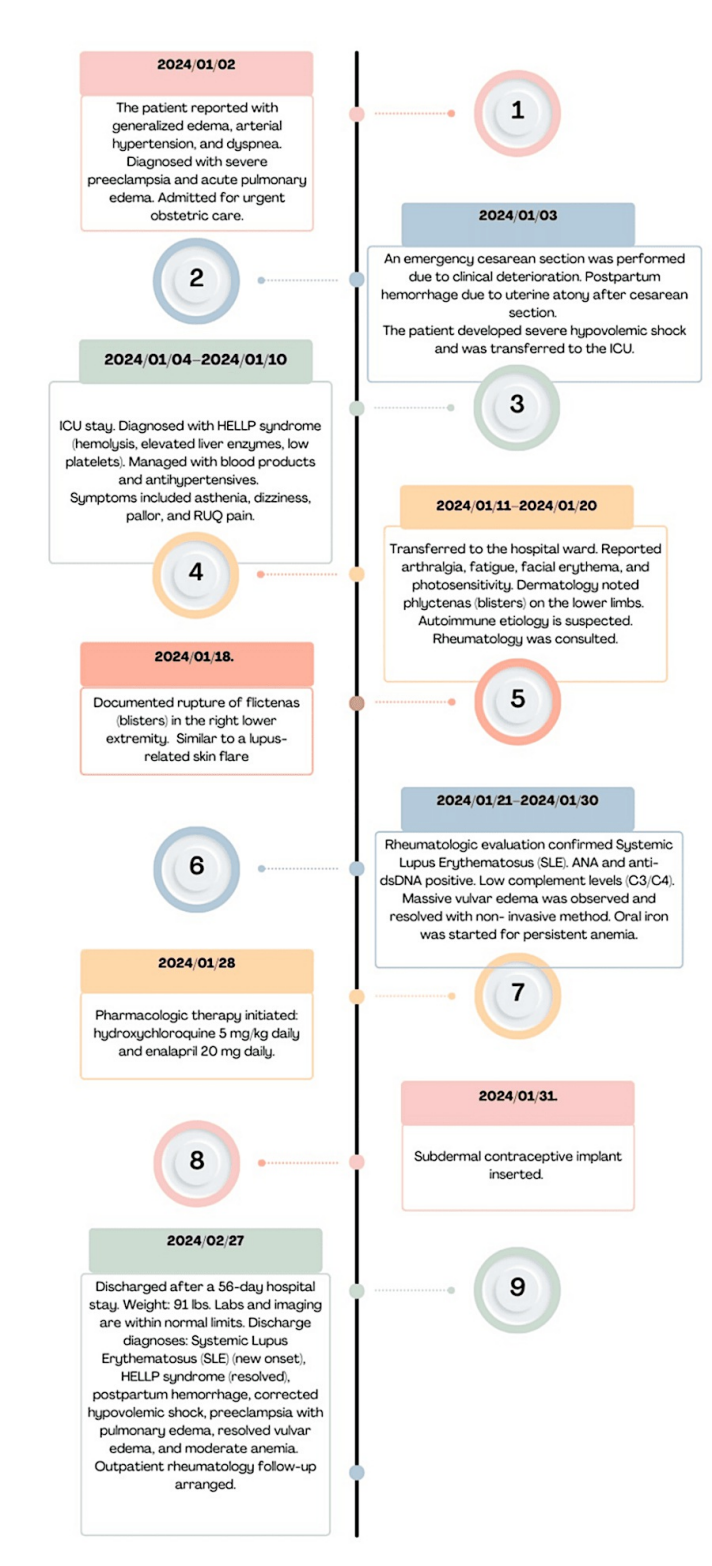
Timeline of Patient's Events Chronological order of events experienced by the patient.

The patient was hospitalized for 56 days and discharged in stable condition on hydroxychloroquine (HCQ) and enalapril. Prior to discharge, a subdermal contraceptive implant was placed, and a follow-up with rheumatology was arranged. Figure 3 shows a timeline summarizing the patient's events.

The patient provided written consent for all images used in this article.

## Discussion

In this report, a case of pregnancy complicated by SLE and other rare manifestations such as severe preeclampsia, HELLP syndrome, and LN is discussed. LN is a variation of lupus that targets the kidneys and can increase the chance of preeclampsia and other APOs [8]. LN increases the chances of morbidity and mortality in patients, with 20% of those with LN advancing to end-stage renal disease (ESRD). LN affects over 50% of patients diagnosed with SLE [9]. Severe preeclampsia affects 10-20% of pregnancies, and HELLP syndrome occurs in approximately 0.1-0.2% of pregnancies [10]. The case presented here is unusual not only due to the third-trimester onset of SLE but also due to the concurrent presence of discoid lesions, LN, HELLP syndrome, and massive vulvar edema in a primigravida adolescent. The combination of these conditions, coupled with SLE, has not been previously reported in indexed literature to our knowledge. A similar case report by Hill et al. describes a pregnancy where complications were misdiagnosed as HELLP or thrombotic thrombocytopenic purpura, which were later confirmed to be lupus flares [11].

A modified B-Lynch suture technique was successfully used, with a reported 95.2% efficiency rate for the management of postpartum hemorrhage [12]. The patient in this case was prescribed hydroxychloroquine at 5 mg/kg/day after discharge, which is well documented to be safe and effective for pregnant and postpartum SLE patients [13]. The use of HCQ throughout the pregnancy journey reduces lupus flares, which suggests the earlier usage of HCQ may have led to a faster recovery in this case.

The complex presentation of SLE in this case was diagnosed during the third trimester of pregnancy. Given the challenges associated with identifying SLE, the patient was not diagnosed with SLE until two weeks after being admitted to the ICU. The patient exhibited signs of a multisystemic autoimmune flare and had symptoms that were initially attributed to pregnancy and severe preeclampsia. Patients with HELLP syndrome and preeclampsia often encounter symptoms that are also key in identifying LN, such as hypertension, thrombocytopenia, and renal dysfunction [14]. The presence of discoid lesions and their massive vulvar edema was significant in prompting further evaluation. Although vulvar edema is rare, it has been previously reported in the context of systemic inflammation and lupus flares, particularly in combination with protein loss and hypoalbuminemia [15]. In order to diagnose SLE, considering the pathophysiological presence does not distinguish the condition from preeclampsia or HELLP syndrome, biomarkers must be used.

Diverse autoimmune antibodies like anti-dsDNA, anti-La/SSB, anti-Ro/SSA, anti-C1q, and antiphospholipid antibodies (aPL) are immunological signatures of SLE. Certain predictors of APOs in patients with SLE include antibody titers, angiogenic and anti-angiogenic biomarkers, miRNA profiles, inflammatory cytokines, proteomic markers, and metabolic biomarkers [16]. Laboratory findings in this case showed low complement levels, positive anti-dsDNA antibodies, proteinuria, and anemia, fulfilling American College of Rheumatology (ACR) diagnostic criteria for SLE. Hill et al. emphasized the importance of testing for SLE biomarkers in pregnant patients with comorbidities to prevent delayed recognition [11]. The infant was delivered at a gestational age of 28 weeks with respiratory distress and a low birth weight, which are common consequences of late preterm birth and maternal lupus. Although both the mother and infant stabilized, the infant has a greater likelihood of facing neurodevelopmental and immunological risks associated with maternal and SLE exposure [17]. Women affected by SLE have about a 33% chance of having anti-Ro/SSA and anti-La/SSB antibodies present. For women with these antibodies present, 10% will have fetuses with neonatal lupus (NL). NL can impact the cutaneous or cardiac regions. Congenital complete heart block (CCHB) may also arise in women with these antibodies with a 1-2% likelihood. This chance increases to 17% if the mother has previously given birth to a child with CCHB [13]. Long-term pediatric follow-up will be crucial for early intervention if complications arise. Additionally, infants born to mothers with active SLE flares are likely to experience developmental risks and require admission to the NICU; hence, follow-ups are important.

During the patient's postpartum period, antihypertensive and immunosuppressive therapies were initiated, including angiotensin-converting enzyme (ACE) inhibitors. ACE inhibitors are used to treat hypertension and prevent renal impairment, along with other ailments, in patients with lupus. ACE inhibitors are not appropriate for patients during pregnancy, as they have been found to increase the chances of fetal malformations and mortality [18]. Despite this, there is no current evidence to suggest that ACE inhibitors are unsafe for postpartum use, as there have been no reported incidents of fetal harm through breastfeeding while on ACE inhibitors. With this in mind, further research is needed to confirm this notion.

Enalapril, captopril, and benazepril are preferred ACE inhibitors during lactation due to the lack of side effects and low excretion into breast milk observed [19]. Other medications that may be used to supplement ACE inhibitors while breastfeeding include hydralazine, nifedipine, and amlodipine. These drugs are considered safe to use, with nifedipine and amlodipine known to be the best calcium channel blockers [20]. In this case, HCQ was first prescribed at the patient's discharge; however, if this medication had been prescribed earlier in the case, it could have reduced SLE flare symptoms and promoted earlier stabilization. HCQ has been shown to minimize the risk of developing pre-eclampsia and flares during and after pregnancy [13]. The patient's postpartum recovery period is particularly important in distinguishing their HELLP syndrome and preeclampsia from atypical hemolytic uremic syndrome (aHUS), as these conditions can present similar laboratory findings as those shown in Table 1. One key feature found in patients with aHUS lies in the postpartum period, where patients will typically experience worsening conditions or failure to recover, while those with HELLP syndrome usually experience rapid recovery [21]. This supports the need for close multidisciplinary collaboration, systematic procedures, and continued care of patients during postpartum who are experiencing complex symptoms such as those presented in this case.

This case exemplifies a rare and complex intersection of SLE, HELLP syndrome, and preeclampsia, all diagnosed during the third trimester of a primigravida adolescent's pregnancy. This underscores the need for greater clinical awareness surrounding SLE and other autoimmune diseases, which serve as the underlying cause for other conditions. Identifying SLE can prevent severe complications and APOs during pregnancy when treated early on. The delayed initiation of HCQ and other immunosuppressants in this case may have contributed to the development of additional afflictions, underscoring the importance of timely management in healthcare settings. Despite the severity of the case, the patient's successful surgical treatment, postpartum stabilization, and favorable neonatal outcomes support current management plans. Aside from follow-up with the rheumatology department, the patient should have a pediatric follow-up to monitor longer-term effects on the child.

## Conclusions

This case demonstrates a rare scenario of new-onset SLE during the third trimester of a primigravida adolescent's pregnancy, which triggered the occurrence of severe preeclampsia, HELLP syndrome, and lupus nephritis. The overlapping features of SLE and common obstetric complications contributed to a significant diagnostic challenge of delayed diagnosis, which increased the risk for APOs. In short, this case accentuates the importance of a timely, thorough diagnostic evaluation due to the complexity of autoimmune disease during late pregnancy and the significant risk posed to the neonate and the mother.

Additionally, timely multidisciplinary care stabilized the patient, emphasizing the importance of early recognition, including targeted treatment of autoimmune diseases and routine use of immunologic biomarkers. These markers, in conjunction with clinical features, facilitate earlier diagnosis and prevent complications through prompt initiation of therapies, especially when atypical symptoms are present. Ultimately, this case reflects the critical need for heightened clinical awareness and reinforces the importance of biomarker-driven diagnostics and patient-centered care in high-risk obstetric circumstances.
